# The potential application of electrophysiological indicators in TMS treatment for MOH

**DOI:** 10.3389/fpain.2025.1689847

**Published:** 2025-11-20

**Authors:** Danqing Xiao, Haochi Han, Huailian Guo, Zhuotong Li, Yang He, Jingqi Ma, Hong Jiang

**Affiliations:** 1 Department of Neurology, Peking University People's Hospital, Beijing, China; 2 Peking University People's Hospital, Beijing, China; 3 Peking University Health Science Center, Beijing, China

**Keywords:** headache, medication overuse headache, electrophysiological indicators, transcranial magnetic stimulation, treatment

## Abstract

Medication Overuse Headache (MOH) can lead to central sensitization (CS), habituation deficits (HD), shortened cortical silent period duration (CSPD), and increased pre-activation levels (PAL), all of which are quantifiable electrophysiological objective indicators related to MOH. Transcranial magnetic stimulation (TMS) is a treatment method for MOH and is primarily divided into three types: single-pulse TMS (sTMS), repetitive TMS (rTMS), and quadruple-pulse TMS (qTMS). Among these, sTMS is convenient for patients of self-administration, qTMS significantly improves the effectiveness of TMS treatment, and rTMS is suitable for widespread use in developing countries. Numerous studies have reported clinical symptom improvements in MOH patients treated with TMS, with statistically significant results. However, only a few studies have observed electrophysiological changes in MOH patients before and after treatment. Whether quantifiable objective indicators can be reversed requires further investigation.

## 1 Introduction

Medication Overuse Headache (MOH) is a secondary headache that develops as a consequence of acute headache medication(s) overuse in headache sufferers. According to the International Classification of Headache Disorders, MOH is defined as headache occurring on 15 or more days/month for at least three months in a patient with a pre-existing headache disorder as a consequence of overuse of acute symptomatic medication (1). It typically results from the overuse of triptans, the combined use of two or more opioid analgesics for at least 10 days per month for more than 3 months, or the use of nonsteroidal anti-inflammatory drugs (NSAIDs) or acetaminophen (paracetamol) for at least 15 days per month for more than 3 months (1). Specific drugs such as triptans, ergotamine, and non-specific drugs like NSAIDs, codeine, tramadol, and pethidine can all lead to MOH (2). Among these, triptans, ergotamine, and opioid analgesics are more likely to trigger MOH, while the risk of developing MOH with NSAIDs and acetaminophen is the lowest (3, 4).

MOH can arise from, but is not limited to chronic migraine (CM) (5, 6) and tension-type headache (TH) (7). The estimated prevalence of MOH in the general population is 1%–2%, while in chronic migraine (CM) patients, approximately 30%–50% suffer from MOH (8, 9). This figure can rise to as high as 80% in tertiary headache treatment centers (10). Patient education, withdrawal of overused medications, preventive therapy (including oral calcitonin gene-related peptide receptor antagonists or botulinum toxin type A), and occipital nerve blocks are the primary treatments for MOH (3, 4, 11, 12). However, some patients do not respond to medication treatment (11), and more than a quarter of MOH patients relapse within the first year (3). Moreover, pharmacological treatments are associated with numerous adverse effects, including gastrointestinal disturbances, dizziness, drowsiness, fatigue, and memory impairment (13). Alternative therapies, particularly physical therapy, hold significant potential for development (11, 13).

Transcranial Magnetic Stimulation (TMS) may be an excellent option for MOH treatment, offering the advantage of avoiding drug adverse effects and demonstrating a favorable safety profile (13–16). The mechanisms of action and methods of use have gradually been revealed in recent research, demonstrating its unique therapeutic potential.

In 1997, Chen first introduced the term “low-frequency” after observing a 20% reduction in the amplitude of motor evoked potentials (MEPs) recorded from muscle following 15 min of 0.9 Hz rTMS applied to the left motor cortex (17). Later, in a 2000 review, Chen formally classified TMS frequencies as “high-frequency” (>5 Hz) and “low-frequency” (≤1 Hz) (18). The distinctions between high- and low-frequency rTMS in headache treatment are summarized in Table 1.

**Table 1:** Differences between high-frequency and low-frequency rTMS in headache treatment.

Classification	Frequency	Direction of cortical modulation	Mechanism	Typical target	Main references
LF-rTMS	≤ 1 Hz	Inhibitory	Analogous to LTD	Right DLPFC Level C evidence for migraine	Lefaucheur 2014 (19)
HF-rTMS	≥ 5 Hz	Excitatory	Analogous to LTP	Left DLPFC Level A evidence for chronic migraine Contralateral M1 Level B evidence for cluster headache	Chen 2000 (18); Lefaucheur 2014 (19); Lefaucheur 2020 (20)

Table 1. Differences between high-frequency and low-frequency rTMS in headache treatment

LF-rTMS(Low-Frequency Repetitive Transcranial Magnetic Stimulation)

HF-rTMS(High-Frequency Repetitive Transcranial Magnetic Stimulation)

LTD(long-term depression)

LTP(long-term potentiation)

R-DLPFC (Right Dorsolateral Prefrontal Cortex)

L-DLPFC(Left Dorsolateral Prefrontal Cortex)

M1(Primary Motor Cortex)

## 2 TMS treatment targets

The selection of TMS targets is crucial for therapeutic efficacy. Commonly used targets include the primary motor cortex (M1), dorsolateral prefrontal cortex (DLPFC), and occipital lobe cortex (OC). These regions have demonstrated therapeutic potential, although underlying mechanisms require further investigation.

### 2.1 Primary motor cortex (M1)

The primary motor cortex, also known as the M1 region in TMS literature, is one of the most commonly targeted areas in TMS applications (21). It is in the precentral gyrus of the cerebral cortex and is primarily responsible for motor functions. The motor hotspot method is the most frequently used M1 localization technique in TMS treatment studies. This method involves delivering single-pulse TMS to the left or right M1 region to activate motor neurons, thereby inducing muscle contractions in the target muscle. The corresponding motor-evoked potentials (MEPs) are recorded via electromyography (EMG), and the M1 target cortical region is determined by identifying the site that consistently produces the most stable MEP. Alternative localization approaches, such as functional magnetic resonance imaging (fMRI)-based reconstruction or anatomical markers like the hand knob region (22), have yielded results similar to those obtained through the motor hotspot method. The analgesic effect of the M1 region primarily depends on its action on endogenous opioid neurotransmitters. Positron Emission Tomography (PET) studies show that M1 stimulation directly enhances the inhibitory system mediated by opioid neurotransmitters (23). In 2018, Andre-Obadia applied 20 Hz rTMS to both the hand and facial areas of M1, and found that stimulation of the hand area of M1 produced significantly greater analgesic effects for facial or upper-limb pain compared with stimulation of the facial M1 area or sham rTMS (24). The analgesic mechanism was suggested to depend on affective-cognitive modulation pathways [such as the anterior cingulate cortex (ACC) and periaqueductal gray (PAG)] rather than on a strictly somatotopic effect.

### 2.2 Dorsolateral prefrontal cortex (DLPFC)

rTMS applied to the left dorsolateral prefrontal cortex (L-DLPFC) at the F3 site of the international 10–20 electrode placement system exerts a broad top-down inhibitory effect along the midbrain-thalamic-cingulate pathway via descending fibers from the prefrontal cortex (25). Consequently, the widespread effects of DLPFC stimulation not only enhance motor cortex function but also modulate affective circuits associated with both pain and depression (26). Two studies directly compared the effects of M1 vs. DLPFC TMS on quantitative sensory testing (QST) measures. During pain treatment with rTMS, high-frequency stimulation is often applied to activate the left DLPFC (27), or low-frequency stimulation to inhibit the right DLPFC (28). This approach may be based on the valence lateralization hypothesis (29), which proposes that the left hemisphere primarily processes positive emotions, whereas the right hemisphere processes negative emotions. Therefore, high-frequency activation of the left DLPFC (27) or low-frequency inhibition of the right DLPFC (28) is selected to modulate emotional and pain-processing networks (20) and thereby achieve an analgesic effect.

### 2.3 Occipital cortex(OC)

Based on gray matter atrophy coordinate network mapping studies (30–32), large-scale data from 1,000 human resting-state functional connectivity (RSFC) cases have been used to link the anatomical coordinates of gray matter volume reduction with brain networks, identifying disease- and symptom-specific brain networks. The reports indicate that functional abnormalities in the brains of patients with Migraine with Aura (MwA) primarily concentrate in the visual regions (33). EEG studies indicate excessive responsiveness of the OC to visual stimulation (34), and positron emission tomography (PET) studies show an increase in activation in the OC in response to light (35). Mapping of the light-sensitive retinal ganglion cells, which relay to the posterior thalamic dura mater sensitive neurons and subsequently project to the OC, provides a neurobiological pathway that may help explain the hyperactivity of visual processing and photophobia in migraine patients (36). Whether OC could serve as novel targets for MOH is a question worth our consideration and further investigation.

## 3 MOH electrophysiological indicators

### 3.1 Amplitude of evoked potentials (Amp), Pre-activation level (PAL), habituation deficit (HD), habituation slope (HS), and central sensitization (CS)

Pre-activation level (PAL) refers to the amplitude (Amp) of the response wave recorded during the first stimulus of an evoked potential. An elevated PAL is defined as central sensitization (CS). In healthy individuals, repeated stimulation of evoked potentials typically results in a gradual decline in response wave amplitude, a characteristic indicative of normal cortical excitability regulation. This adaptive process protects the nervous system from sensory overload and optimizes attentional and memory resources for novel stimuli and this phenomenon is known as habituation. An impairment in this process is referred to as habituation deficit (HD) (37–40).HD is assessed based on the habituation slope (HS), which represents the slope of the change in evoked potential amplitudes or areas (41). Sensory information reaches the cortex through synchronously active thalamic axons, providing a strong excitatory drive to layer 4 (L4) cortical neurons (42). Inefficient thalamocortical drive leads to elevated sensory cortical PAL, resulting in an imbalance in the excitation-inhibition network. This dysfunction can induce hyperresponsiveness of primary sensory cortical neurons, a phenomenon commonly observed in individuals with CM and MOH. A study assessing 29 MOH patients, 64 migraine without aura (MOA) patients, and 42 healthy volunteers (HV) during both attack and interictal phases used N20-HS from median nerve somatosensory evoked potentials (SSEP) to evaluate cortical and subcortical excitability (43). In the MOH group, an increase in response amplitude (Amp) to a small number of repeated stimuli indicates the presence of central sensitization (CS), while the lack of amplitude reduction during subsequent stimulations reflects a habituation deficit (HD). This pattern is similar to the pre-attack phase of episodic migraine (EM), suggesting that the somatosensory cortex has become persistently sensitized. MOH patients appear to be locked in a “sustained attack state,” characterized by hypersensitivity due to high PAL and hyperreactivity due to high HD, which negatively impacts neuronal plasticity (44). This phenomenon may even contribute to widespread cutaneous allodynia (45, 46). Subgroup comparisons in MOH revealed that MOH-NSAIDs patients exhibited increased PAL and the presence of HD, suggesting a potential reduction in inhibitory interneuron function. In contrast, while MOH-triptans patients also showed HD phenomena, there was no increase in PAL, which may be related to the shorter duration of withdrawal headaches in MOH-triptans compared to MOH-NSAIDs patients (47).

### 3.2 Motor evoked potential (MEP), cortical spreading depression (CSD), and cortical silent period duration (CSPD)

Cortical Spreading Depression (CSD) is a slowly propagating depolarization wave involving neurons and glial cells, which can be seen on the electroencephalogram (EEG) as a high-amplitude negative depolarization wave followed by a high-voltage slow wave or a flat suppression state. CSD exhibits the characteristic of spreading: this depolarization wave accompanied by suppression gradually extends to surrounding cortical areas, with the affected regions slowly returning to normal electrical activity after the suppression phase (48). Cortical spreading depression plays a significant role in aura development (49). Stereotactic electroencephalography in a patient with an attack of MwA demonstrated low-voltage suppression initiating in the left mesial occipital cortex and propagating anteriorly at approximately 3 mm/min, corresponding clinically to a contralateral right superior scintillating scotoma and ipsilateral headache. This is the first definitive electrophysiological demonstration of cortical spreading depression occurring during a migraine headache (50). Silent Period (SP) refers to a period of muscle contraction cessation observed on electromyography (EMG) following stimulation of peripheral nerves, the brainstem, or the cortical area. During this phase, the EMG records a pause in muscle contraction, resulting in a period of electrical silence (51). And the Cortical Silent Period Duration (CSPD) is defined as the sustained period of EMG silence recorded in a target muscle following a single-pulse TMS applied to the M1 region during voluntary muscle contraction. This suppression begins after the motor evoked potential (MEP) and persists until voluntary contraction of the target muscle resumes, reflecting intracortical inhibitory neural mechanisms (52). A study (53) have assessed the characteristics of MEP and CSPD in MOH patients using the orbicularis oris muscle, included 9 MOH-triptans, 9 MOH-NSAIDs, 12 MOH-Bi (MOH patients with overuse of two types of medications), 12 EM, and 13 HV. The CSPD duration among (sub)groups showed the following differences: MOA < MOH-triptans < MOH-Bi < MOH-NSAIDs < HV. A negative correlation was observed between the monthly intake of triptans and CSPD in MOH-triptans. Additionally, an earlier study reported that pain-related cortical evoked potential amplitudes in MOH-triptans were higher than in HV (54). Collectively, these findings suggest that triptans may enhance cortical excitability mechanisms. Although both MOH-triptans and MOH-NSAIDs contribute to migraine chronification, their clinical manifestations do not completely overlap. MOH-triptans tend to experience daily headaches resembling migraine, while MOH-NSAIDs are more prone to typical tension-type daily headaches (55, 56). This difference may be related to the fact that CSPD is relatively prolonged in MOH-NSAIDs compared to MOH-triptans, as the interictal CSPD in CM-MOH-NSAIDs was found to be nearly comparable to that in HV (57). Researchers observed 18 patients with MOH who discontinued triptans and NSAIDs without using preventive medications. After 3 weeks, they re-evaluated CSPD. Among the patients, 10 MOH-Bi patients showed a significant reduction in CSPD after discontinuing the medication, while 8 MOH-triptans patients had similar CSPD before and after discontinuation. Due to the lack of a group of patients who only discontinued NSAIDs, it remains to be determined through further research whether the shorter duration of withdrawal headache in MOH-triptans patients compared to MOH-NSAIDs can be explained by CSPD change.

### 3.3 Multisensory evoked potential integration

Clinical sensory processing impairments, such as photophobia and phonophobia, are positively correlated with headache intensity (58). Visual or auditory stimuli can trigger migraine attacks in 50%–75% of patients (59). Moreover, migraine patients may experience visual and auditory discomfort even during pain-free periods, which can intensify during attacks (60, 61). A coherent, distinct, and stable perceptual experience arises from the ability to integrate shared sensory information across different modalities (62–64). Multisensory integration occurs when stimuli from different sensory systems are temporally or spatially linked (65–67). Cross-modal illusions serve as a valuable paradigm for assessing how multisensory integration influences perception (68, 69). A well-known example is the sound-induced flash illusion (SIFI), which includes the fission illusion (Fis) and the fusion illusion (Fus), both of which are associated with cross-modal variations in visual cortical excitability. Transcranial electrical stimulation that enhances occipital cortical excitability, or occipital infarcts, can disrupt SIFI, whereas right parietal cortical lesions may cause preserved or even enhanced SIFI effects (70). The Fus and Fis effects represent dissociable phenomena, with Fis being more closely linked to cortical excitability states (70–72). A study investigating SIFI levels in 63 patients with chronic migraine (CM), including 52 medication-overuse headache (MOH) patients (83%) and 24 HV. The results indicated that MOH patients—particularly those in the MOH-triptans subgroup—exhibited excessive excitability in the visual cortex. This disrupted the expected low SIFI effect seen in HV, leading to increased signal resolution in the identification of flash stimuli, potentially due to reduced cortical inhibition within the primary visual areas. These findings suggest that medication-induced maladaptive neuroplasticity may contribute to visual cortical hyperexcitability, increasing susceptibility to MOH triggers. Further exploration of SIFI mechanisms in MOH may help design more targeted preventive treatments.

## 4 Research status of different TMS paradigms

TMS has been applied in the treatment of MOH, with commonly used types including sTMS (Single-pulse Transcranial Magnetic Stimulation),rTMS (Repetitive Transcranial Magnetic Stimulation), and iTMS (Intermittent Theta Burst Stimulation). Research specifically investigating the therapeutic effects of sTMS, rTMS, and qTMS in MOH populations is limited. Most studies have primarily focused on migraine and chronic headache patients, although some have involved MOH. More targeted research on MOH may be needed in the future.

### 4.1 Single-pulse TMS (sTMS)

sTMS temporarily disrupts brain activity by interrupting CSD, thereby reducing the occurrence of migraine aura (73). It influences neurotransmitter release by decreasing excitatory glutamate and increasing inhibitory gamma-aminobutyric acid (GABA), which lowers neuronal excitability and helps reduce the frequency and severity of migraines (74, 75). sTMS can also modulate the spontaneous activity of third-order thalamic neurons and trigeminovascular activity induced by C-fibers (73). The first long-term study on sTMS at occipital cortex for adults with CM and high-frequency episodic migraine (HFEM) (76) (≥8 headache days per month) included more than half of the patients with MOH and demonstrated significant efficacy. The proportion of MOH patients decreased from 52% (*N* = 79/153) at baseline to 19% (*N* = 29/153) at the third month and 8% (*N* = 7/87) at the twelfth month (77). Participants underwent self-administered TMS therapy 2–3 times daily for three months. Compared to baseline, the median HIT-6 score decreased by 4 points at the twelfth month. The proportion of patients with severe headache-related disability dropped from 93% at baseline to 63% at both the third and twelfth months. Regardless of MOH status or treatment resistance level, 45% of patients experienced long-term headache improvement. When assessing treatment sustainability using HIT-6 score changes, patients who showed significant score reductions were more likely to continue treatment for 12 months, indicating their recognition of the positive effects of sTMS (77).

### 4.2 Repetitive TMS (rTMS)

The effects of rTMS on cortical excitability can persist for an extended period, with the direction of modulation primarily depending on the stimulation frequency (78). Since various treatment protocols can be designed to target different pathophysiological aspects of MOH, rTMS theoretically allows for personalized treatment tailored to individual patient needs (79).High-frequency rTMS (HF-rTMS) may promote dendritic spine structural remodeling by reshaping postsynaptic scaffolding proteins and modulating synaptic GABAergic activity. Additionally, it can increase endorphin levels in migraine patients (15). In 2014, Misra (15) studied the effects of left frontal HF-rTMS treatment in 94 migraine patients, including 22 MOH patients. Among them, 56 patients received real stimulation, while 38 received sham stimulation. A reduction in N20 amplitude was found to be associated with decreased headache frequency and severity over one month. Compared to the sham group, the real stimulation group experienced significant HD improvement, which was associated with reduced headache severity but not frequency. That same year, Conforto (14) reported negative results for HF-rTMS targeting the left DLPFC. He randomized 18 CM patients [of whom 14 (77.78%) had MOH] into real and sham stimulation groups (1:1 ratio). Over eight weeks, patients underwent 23 rTMS sessions, with headache days assessed through a headache diary. The study found no significant benefits of HF-rTMS-L-DLPFC and suggested that M1 might be a more promising target than DLPFC. In 2016, Indian researcher Kalita (80) investigated the differences between single-session and three-session HF-rTMS-L-DLPFC for chronic headache treatment. The study included 82 participants, comprising CM and chronic tension-type headache (CTTH) patients, of whom 36 had MOH. After treatment, no significant differences were observed in headache frequency reduction between CM patients with and without MOH at 1, 2, and 3 months post-treatment. Among 10 CTTH patients and 6 mixed chronic daily headache (CDH) patients (including 5 MOH patients), headache frequency showed improvement after rTMS, but without statistically significance. Three years later, Granato (81) conducted a study on 14 MOH patients (10 overusing triptans & NSAIDs, 4 overusing NSAIDs only), administering 20 Hz HF-rTMS-L-DLPFC treatments over two consecutive weeks. The study assessed headache days, duration, intensity, medication use, and disability levels. The results indicated a strong placebo effect, with headache days reduced by 45.5% in the treatment group and 40% in the sham group, failing to confirm the therapeutic efficacy of HF-rTMS-L-DLPFC.

### 4.3 Quadruple-pulse rTMS (qTMS)

Quadruple-pulse transcranial magnetic stimulation (qTMS) delivers four monophasic magnetic pulses at variable interstimulus intervals (ISI), achieving excitatory effects when ISI < 10 ms and inhibitory effects when ISI > 30 ms on the underlying cortex. Initially, studies on qTMS focused on M1. Researchers recruited 10 HV and assessed motor cortical excitability and plasticity by measuring the peak MEP-Amp from the relaxed right first dorsal interosseous (FDI) muscle. Their findings demonstrated that qTMS induces long-lasting excitability and plasticity changes in M1, suggesting its potential preventive effects on MOH attacks (82, 83). Following qTMS treatment, MEP measurements were conducted every 5 min for 30 min and then every 15 min for 180 min while maintaining a constant output intensity. Results showed that qTMS at short ISIs (1.5–10 ms) enhanced MEPs for over 75 min, resembling long-term potentiation (LTP), whereas long ISIs (30–100 ms) suppressed MEPs, mimicking long-term depression (LTD). Thus, qTMS can bidirectionally modulate synaptic plasticity by altering ISI. Given that sTMS has demonstrated acute analgesic effects on MwA in the visual cortex of EM patients (84) and preventive effects (77), Viganò et al. were the first to apply qTMS to the visual cortex of patients with chronic migraine and medication-overuse headache (CM-MOH). Their inhibitory protocol (qTMS-I) involved delivering 4-pulse sequences every 5 s with an ISI of 50 ms for 30 min (totaling 1,440 pulses). This protocol reduced VEP-Amp and HS in HVs (85). During a one-month treatment period (twice per week), CM-MOH patients also exhibited reductions in VEP-Amp and HS, with the therapeutic effect more pronounced 3 h post-stimulation than immediately after treatment. This qTMS-induced reduction of visual cortical CS led to a nearly 50% reduction in monthly headache days in CM-MOH patients, with HIT-6 score improvements persisting for one month post-treatment. Additionally, half of the patients (*n* = 6) reverted to an EM pattern (ICHD-3 1.1) (86), allowing for the reintroduction of preventive medications (87). However, severe headache days (Grade 3 intensity), acute medication intake, MIDAS, STAI, and BDI scores did not show significant improvements. A single session of occipital qTMS-I only caused a transient VEP-Amp reduction, with no significant effects on other VEP parameters, suggesting that repetitive rTMS sessions are necessary to induce long-lasting plasticity changes in sensory cortices (88). Building on previous studies (83, 89) and meta-analyses (90), researchers designed a simulated excitatory protocol (qTMS-E), which delivered pre-stimulation pulses to functional MRI (fMRI) of the occipital region V2 and V3 areas every 5 s with an ISI of 50 ms for 10 min, followed by stimulation of V1 area every 5 s with an ISI of 30 ms for 30 min. In the six patients who reverted to EM, VEP-HD significantly improved one month after the qTMS-I treatment phase (39, 91, 92).

## 5 Placebo effects of rTMS

Placebo-controlled comparisons are essential for evaluating the efficacy of interventions in RCT (randomized controlled trial). The placebo effect arises from various factors, including but not limited to the clinician's demeanor during treatment (enthusiastic, indifferent, or neutral) (93, 94), the patient's awareness of the research process and trial details, and the subjective perception of symptoms, particularly pain. In some studies, placebo interventions have demonstrated clinically relevant response rates of ≥50% (75, 95, 96). A study by Huang et al. (97) on the placebo effects of non-pharmacological therapies found that sham rTMS was significantly more effective than sham CBT(Cognitive Behavioral Therapy), sham nVNS (non-invasive Vagus Nerve Stimulation), sham tDCS (transcranial Direct Current Stimulation), and sham acupuncture in reducing headache days, likely due to the high procedural similarity between sham and real rTMS (14, 75, 81, 98–103). Notably, one study even reported that sham rTMS yielded a higher response rate in reducing headache days than the active intervention, contradicting the classical RCT interpretation that “the most effective treatment is the one with the greatest specificity beyond placebo” (104). This paradox may be attributed to the small sample size in sham rTMS trials, as smaller sample sizes tend to exaggerate treatment effects (105–107). These findings underscore the need for large-scale RCTs on rTMS for MOH, incorporating biomarker-based objective quantification. Such studies would not only elucidate the pathophysiology of MOH but also enhance our understanding of TMS mechanisms, providing strong clinical evidence to support its application in practice.

## 6 Discussion

Research on TMS for MOH remains limited, and many findings must be extrapolated from studies on CM or EM. The effectiveness of the L-DLPFC target is still debated, while MOH studies targeting the occipital lobe are nearly nonexistent. Additionally, few studies have used objective electrophysiological biomarkers as primary outcome measures, providing an important direction for future MOH treatment research design. Several factors may contribute to variability in the efficacy of TMS for MOH (14, 102, 108): (i) Differences in neuroplasticity and excitability alterations between MOH-NSAIDs and MOH-triptans patients may lead to distinct treatment responses (109–111). (ii) Lack of strict adherence to guidelines (112), including variations in stimulation timing (80, 90), target selection, and stimulation protocols across studies (113). (iii) Inconsistent study designs and measurement parameters, such as differences in baseline characteristics, statistical methods, small sample sizes, uneven group distributions, and lack of long-term follow-up (114, 115). (iv) Placebo effects cannot be ruled out, as the clinician's approach, the participant's understanding of the study, and the inherent placebo effect of device-based therapies (which is generally greater than that of oral medications) may influence outcomes (81, 93, 94, 116).

In the future, multimodal measurements of electrophysiological indicators such as Amp, PAL, HD, HS, CS, MEP, CSD, CSPD, and SIFI, along with TMS-induced high-density electroencephalographic responses (TMS-EEG), will help unravel the underlying mechanisms of CS and HD in MOH patients. These measurements will also allow for more accurate determination of the effects of TMS on the entire brain or other brain regions with abnormal electrical activity, thereby identifying more effective treatment targets and efficacy evaluation indicators for MOH.

## 7 Conclusion

Future research on TMS treatment for MOH will place greater emphasis on individualized therapy, with electrophysiological assessments providing an objective evaluation metric for this approach. Currently, most studies on TMS treatment for MOH rely on subjective efficacy evaluations based on clinical manifestations, such as pain diaries and pain scales. There is limited research using electrophysiological indicators to assess the efficacy of TMS in treating MOH. By combining objective electrophysiological metrics with subjective clinical symptom indicators, researchers can better understand how TMS modulates neural circuits to achieve pain relief. This will help optimize the clinical application of TMS and enhance our ability to harness this promising treatment method more effectively.
